# Correction: Putting perception into action with inverse optimal control for continuous psychophysics

**DOI:** 10.7554/eLife.100253

**Published:** 2024-06-03

**Authors:** Dominik Straub, Constantin Rothkopf

**Keywords:** Human

Straub D, Rothkopf CA. 2022. Putting perception into action with inverse optimal control for continuous psychophysics. *eLife* **11**:e76635. doi: 10.7554/eLife.76635.

Published 29 September 2022

After publication, we have found an error in the implementation of the adjustment of the psychophysical thresholds used to obtain single-interval sensory uncertainties from two-interval forced-choice data. While we write in Appendix 4 that "we divided the estimates of the perceptual uncertainty by √2 to obtain single-interval sensory uncertainties", our code used a multiplication instead of the division. This error does not affect the models applied to the tracking data, but it does affect how they compare against the classical psychophysical measurements. Fixing the bug did not qualitatively change any of the conclusions of the paper, but some numbers in the results need to be updated. Specifically, this affects the numbers reported in the result subsection "Continuous psychophysics" as detailed below and the two-interval forced choice estimates (green lines) in Figure 4A.

Corrected text:

The average factor between the posterior mean perceptual uncertainty in the continuous task and the 2IFC task in the five higher contrast conditions is 2.39 for the subjective actor, while it is 3.46 for the KF. Only in the lowest contrast condition, it increases to 4.40 and 5.87, respectively.

Original text:

The average factor between the posterior mean perceptual uncertainty in the continuous task and the 2IFC task in the five higher contrast conditions is 1.20 for the subjective actor, while it is 1.73 for the KF. Only in the lowest contrast condition, it increases to 2.20 and 2.93, respectively.

The corrected Figure 4 is shown here:

**Figure 1:**
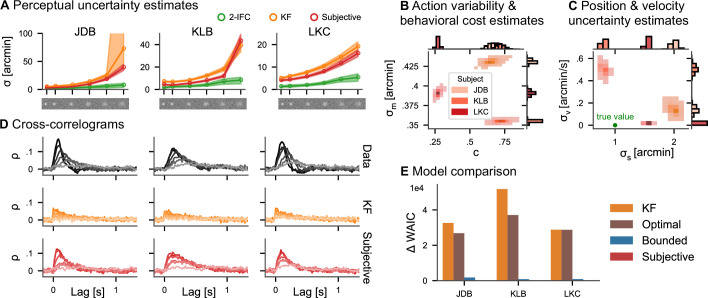


The originally published Figure 4 is shown for reference:

**Figure 2:**
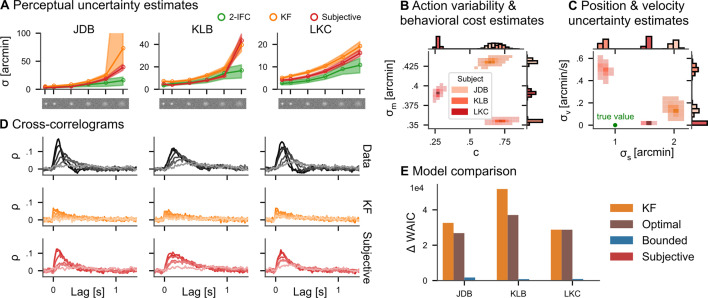


The article has been corrected accordingly.

